# The pathogenic c.1171A>G (p.Arg391Gly) and c.2359G>A (p.Val787Ile) ABCC6 variants display incomplete penetrance causing pseudoxanthoma elasticum in a subset of individuals

**DOI:** 10.1002/humu.24498

**Published:** 2022-11-15

**Authors:** Flora Szeri, Agnes Miko, Nastassia Navasiolava, Ambrus Kaposi, Shana Verschuere, Beatrix Molnar, Qiaoli Li, Sharon F. Terry, Federica Boraldi, Jouni Uitto, Koen van de Wetering, Ludovic Martin, Daniela Quaglino, Olivier M. Vanakker, Kalman Tory, Tamas Aranyi

**Affiliations:** ^1^ Department of Dermatology and Cutaneous Biology, The Sidney Kimmel Medical College, and The PXE International Center of Excellence in Research and Clinical Care Thomas Jefferson University Pennsylvania Philadelphia USA; ^2^ Institute of Enzymology Research Centre for Natural Sciences Budapest Hungary; ^3^ Department of Biochemistry Semmelweis University Budapest Hungary; ^4^ MTA‐SE Lendület Nephrogenetic Laboratory Budapest Hungary; ^5^ 1st Department of Pediatrics Semmelweis University Budapest Hungary; ^6^ PXE Consultation Center, MAGEC Nord Reference Center for Rare Skin Diseases Angers University Hospital Angers France; ^7^ Department of Programming Languages and Compilers Eötvös Loránd University Budapest Hungary; ^8^ Center for Medical Genetics Ghent University Hospital Ghent Belgium; ^9^ PXE International District of Columbia Washington USA; ^10^ Department of Life Sciences University of Modena and Reggio Emilia Modena Italy; ^11^ Interuniversity Consortium for Biotechnologies (CIB) Trieste Italy; ^12^ Department of Molecular Biology Semmelweis University Budapest Hungary

**Keywords:** calcification, genetic diagnosis, incomplete penetrance, pseudoxanthoma elasticum, pyrophosphate, rare disease

## Abstract

ABCC6 promotes ATP efflux from hepatocytes to bloodstream. ATP is metabolized to pyrophosphate, an inhibitor of ectopic calcification. Pathogenic variants of ABCC6 cause pseudoxanthoma elasticum, a highly variable recessive ectopic calcification disorder. Incomplete penetrance may initiate disease heterogeneity, hence symptoms may not, or differently manifest in carriers. Here, we investigated whether incomplete penetrance is a source of heterogeneity in pseudoxanthoma elasticum. By integrating clinical and genetic data of 589 patients, we created the largest European cohort. Based on allele frequency alterations, we identified two incomplete penetrant pathogenic variants, c.2359G>A (p.Val787Ile) and c.1171A>G (p.Arg391Gly), with 6.5% and 2% penetrance, respectively. However, when penetrant, the c.1171A>G (p.Arg391Gly) manifested a clinically unaltered severity. After applying in silico and in vitro characterization, we suggest that incomplete penetrant variants are only deleterious if a yet unknown interacting partner of ABCC6 is mutated simultaneously. The low penetrance of these variants should be contemplated in genetic counseling.

*ABCC6* encodes a transmembrane ATP‐binding cassette protein primarily expressed in the liver, less in the kidney and the gastrointestinal tract (Arányi et al., 2005; de Boussac et al., 2010). The basolateral protein promotes the efflux of intracellular ATP to the bloodstream (Jansen et al., 2013, 2014). ATP is subsequently metabolized to pyrophosphate (PPi), a major inhibitor of ectopic calcification (Back et al., 2019; Dedinszki et al., 2017). Biallelic loss‐of‐function (LOF) variants of ABCC6 cause pseudoxanthoma elasticum (PXE) (Le Saux et al., 2000; Ringpfeil et al., 2000), a slowly progressing disease (Favre et al., 2017), characterized by soft tissue calcification with dermatologic, ocular, and cardiovascular manifestations, including vascular calcification, intermittent claudication, and higher incidence of cardiovascular events (De Vilder et al., 2018; Larusso et al., 2010). The prevalence of PXE is ~1/25,000–50,000 (De Vilder et al., 2018; Larusso et al., 2010) and over 350 pathogenic variants were reported in the several thousands of patients recruited worldwide (Boraldi et al., 2021; Larusso et al., 2010; Legrand et al., 2017; Moitra et al., 2017; Saeidian et al., 2022).

In monogenic diseases not necessarily all individuals harboring a disease‐causing genotype develop the clinical phenotype (Ahluwalia et al., 2009; Moore et al., 1993; Venturini et al., 2012), a phenomenon termed incomplete penetrance (IP). Some mutations have a penetrance below 5%, making genetic counseling challenging (Mikó et al., 2021; Minikel et al., 2016). Although IP is well‐known in autosomal dominant diseases (Ahluwalia et al., 2009; Venturini et al., 2012), it was just recently acknowledged in recessive disorders (Israel et al., 2017), such as in cystic fibrosis (*CFTR*) (Mikó et al., 2021), and NPHS2‐associated Steroid‐Resistant Nephrotic Syndrome (Tory et al., 2014).

IP can be assessed by comparing patient cohorts to reference populations, now available thanks to worldwide sequencing efforts (Wright et al., 2019). Using a recently developed algorithm (Mikó et al., 2021) applying differences in allele frequencies (AF) of the patient and reference populations, the penetrance of a pathogenic variant can be calculated.

To improve genetic counseling and shed more light on disease mechanism, in this study, we investigated whether pathogenic *ABCC6* alleles exhibit incomplete penetrance in a phenotypically and genotypically well‐characterized large European PXE patient cohort.

We first compiled an anonymized cohort of clinically (age and Phenodex score, Pfendner et al., 2007) and molecularly diagnosed PXE patients (Supporting Information: Methods). Altogether 589 probands with European ancestry from three countries (Belgium, France, and Italy, Boraldi et al., 2021; Vanakker et al., 2008) were collected (Supporting Information: Table 1A). Only patients of known ethnic origin, familial relationship, and with biallelic *ABCC6* variants considered to be disease‐causing at the time of diagnosis were included in our cohort. The cohort contained 217 distinct variants including missense, nonsense, frameshift variants, and large deletions (Figure 1a and Supporting Information: Table S1b). (Nitschke et al., 2012; Omarjee et al., 2019; Uitto et al., 2010). Of note, due to the high clinical heterogeneity of PXE some patients with mild phenotype might be missed and the cohort may not be fully representative of the variant distribution.

**Figure 1:**
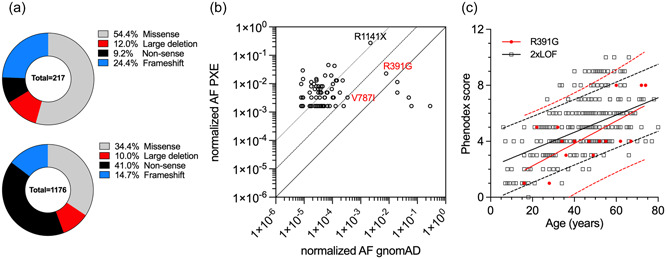
Enrichment and severity of p.R391G pathogenic variant in the European PXE patient population. (a) Distribution of frequency of mutation types in the European patient cohort of 589 individuals. The upper panel depicts the ratio of each allele variant falling into the four categories. The lower panel shows the actual allele frequency of variants in the cohort. (b) Enrichment of sequence variants in the patient population relative to the European non‐Finnish population of gnomAD database. Each dot represents the AF of an individual sequence variant in the two databases in a logarithmic scale. Solid, dashed, and punctuated lines represent 1x, 10x, and 100x enrichments, respectively. The incomplete penetrant p.R391G and p.V787I and the most frequent p.R1141* are also indicated. The sequence variants not represented on this logarithmic plot because they are absent from the gnomAD database are shown in Supporting Information: Table S1C). (c) Relationship between Phenodex score and age. Open squares represent the sum of Phenodex scores of individual patients with two truncating pathogenic variants. Red circles represent patients with one p.R391G pathogenic variant (a). Black and red lines represent linear regression of the patients with 2xLOF and p.R391G, respectively. Dotted lines matching the line colors show 95% confidence intervals for the linear regression (Y_2xLOF_ = (0.062 ± 0.007) * X + 2.182 ± 0.323; Y_R391G_ = (0.080 ± 0.024) * X + 0.633 ± 1.175; *R* ^2^_2xLOF_ = 0.551, *R* ^2^_R391G_ = 0.452).

To determine the frequency of *ABCC6* sequence variants in PXE patients and the general population the allele variants of our cohort underwent several filtering steps (Supporting Information: Methods). As a result, only 616 alleles remained from the initial 589 patients. Subsequently, an enrichment analysis was performed by comparing the AF of the remaining variants of the patient cohort and the European non‐Finnish population of the gnomAD database (https://gnomad.broadinstitute.org) (Karczewski et al., 2020) (Figure 1b and Supporting Information: Table S1c). This control population is overrepresented for people of Scandinavian origin, however, this did not bias our previous studies (Mikó et al., 2021). Most of the 616 *ABCC6* alleles in the patient cohort are present as a single cluster after the comparison, far above the 1 times enrichment threshold, demonstrating their pathogenicity and suggesting full penetrance (Mikó et al., 2021). In this cluster, c.1552C>T (p.Arg518*), c.1553G>A (p.Arg518Gln), c.1132C>T (p.Gln378*), c.3736‐1G>A, and c.3412C>T (p.Arg1138Trp) were represented by AF>1.5% in the patient cohort with *n* = 28, 26, 18, 12, and 12 alleles, respectively. Importantly, the vast majority of the previously reported pathogenic variants were indeed significantly enriched in the patient cohort relative to the general population.

Strikingly, c.3803G>A (p.Arg1268Gln), c.793A>G (p.Arg265Gly), c.3507‐3C>T, c.2836C>A (p.Leu946Ile), c.346‐6G>A were not enriched in the patient population (below the one times enrichment threshold). Thus these variants are more frequent in the general population than in the patient population, and most likely represent benign, non‐pathogenic variants that were probably mistakenly identified previously as disease‐causing. We excluded these variants from further analysis.

Two additional outliers of the cluster of the pathogenic variants were identified. The first was c.3421C>T (p.Arg1141*), which had a high AF both in the general and the patient population (124‐fold enrichment, Supplementary Table 1d) accounting for almost 1/3 of all PXE‐causing mutations. The second was c.1171A>G (p.Arg391Gly), which had an unexpectedly high AF in the general population (0.78%) and showed a small but significant enrichment amongst patients (2.89‐fold enrichment; *p* = 5 × 10^‐4^). This relatively low enrichment raised the possibility that this variant was incompletely penetrant.

To confirm this and identify additional incompletely penetrant ABCC6 variants, in the second part of the analysis, we performed the IP test on the significantly enriched alleles. The algorithm compared the enrichment of each variant in the patient versus general population to the combined enrichment of fully penetrant complete LOF variants. Seventy‐five missense sequence variants were included in this analysis, as the others were not represented in gnomAD. Only variants leading to truncated ABCC6 were considered to be LOF. The penetrance was calculated as the ratio of the two distinct fold‐enrichments, as described formerly (Mikó et al., 2021). In this analysis, we identified two variants as incompletely penetrant: c.2359G>A (p.Val787Ile) and c.1171A>G (p.Arg391Gly). The c.2359G>A (p.Val787Ile), despite its low frequency, was enriched significantly in the PXE cohort relative to the general population (9.53‐fold; *p* = 0.02). Nevertheless, this enrichment was much less prominent than that of the LOF pathogenic variants (Supporting Information: Table 1d), demonstrating potential IP of the c.2359G>A (p.Val787Ile) (6.5%; *p* < 5.1 × 10^−8^), suggesting that in >90% of the patients who are compound heterozygous for this variant and have a completely penetrant variant on the other allele, PXE will likely not develop. For c.1171A>G (p.Arg391Gly), the second most frequent missense variant in our patient cohort (17/589 patients), we calculated 1.98% penetrance (*p* < 2.6 × 10^−118^). This suggests that only two c.1171A>G (p.Arg391Gly) heterozygous carriers would develop PXE out of 100 individuals provided they carry a completely penetrant pathogenic variant on the other allele.

We used the US International PXE cohort (Saeidian et al., 2022), similar in size (478 patients) as the European cohort, to validate the AF and enrichment of the IP pathogenic variants. None of the US patients harbored the c.2359G>A (p.Val787Ile) variant. In contrast, the cohort contained 11 patients being compound heterozygous for the c.1171A>G (p.Arg391Gly). In five additional patients (not included in further analyses) the c.1171A>G (p.Arg391Gly) variant was the only detected pathogenic ABCC6 variant without any reported pathogenic variant identified on the other allele (AF = 2.34%). The enrichment of the c.1171A>G (p.Arg391Gly) allele in the US patient population was 2.98 (*p* = 0.0045). Although familial relationship and ethnicity of the US patients were not available, making calculations only approximate, these data correspond to the AF and enrichment observed in the European PXE cohort.

To assess phenotype severity of IP variants we compared Phenodex scores of patients bearing the IP variant with that of patients having two LOF alleles (2x LOF). To increase statistical power, here we included all patients of the European cohort. As expected, Phenodex scores reflected disease progression as a function of age (Figure 1c). The c.2359G>A (p.Val787Ile) variant could not be analyzed due to low patient numbers. Linear regression analysis revealed no significant difference between patients with the c.1171A>G (p.Arg391Gly) variant and patients with two LOF alleles, indicating similar disease severity and progression for the IP variant.

To shed light on the genetic mechanisms of IP in PXE, in the following, we tested whether the observed IP of c.1171A>G (p.Arg391Gly) could be explained by an effect on splicing, which frequently causes reduced penetrance (Cogan et al., 2012; Rave‐Harel et al., 1997), or by its obligate association with another pathogenic variant(s) within the *ABCC6* gene either in *trans* or *cis* position. Although the position at c.1171 is in close proximity to the 3′ end of exon 9 (n‐5 bp), alternative splice site predictor, (Wang & Marín 2006) indicated no likely altered splicing event. As the *ABCC6* is primarily expressed in the liver, a potential splicing alteration in the presence of c.1171A>G (p.Arg391Gly) could not be further investigated due to the lack of patient tissue biopsy. However, we could test the putative association of p.R391G with any specific pathogenic variant of *ABCC6* on the other allele (*trans* position). We found that from the initial 17 European patients with c.1171A>G (p.Arg391Gly) only three had missense pathogenic *trans* variants. All other c.1171A>G (p.Arg391Gly) alleles of the patients associated with LOF variants on the other allele. However, we found no significant difference (*p* = 0.17) in the association of c.1171A>G (p.Arg391Gly) to LOF compared to missense variants in the *trans* position. The above analysis ruled out the specific association of any missense variant on the other (*trans*) allele in PXE patients carrying c.1171A>G (p.Arg391Gly). Next, we postulated that the R391 residue is involved in a pivotal intramolecular interaction. We assumed that this putative intramolecular interaction is altered by the substitution of arginine by glycine only if another residue is also mutated in the same ABCC6 molecule, requiring an associated second alteration of the same allele (*cis* position) along with c.1171A>G (p.Arg391Gly). This hypothesis is supported by the conservation of the R391 residue (Supporting Information: Figure S1a), and by homology models of ABCC6. The Arg391 and its flanking residues encoded by exons 9 and 10 are well conserved throughout evolution (Supporting Information: Figure S1a), indicating the importance of this region for protein function (Clustalw) (Thompson et al., 1994). Furthermore, Arg391 is also conserved in other members of the ABCC family (ABCC1, ABCC3, and ABCC4), while it is replaced by the similarly charged lysine in ABCC2 and ABCC7 (CFTR) (Ran et al., 2018) (Figure 2b). Arg391, according to the most recent homology models built on the crystal structures of bovine ABCC1 (Charbel Issa et al., 2020) (Szeri et al., 2021), is located in a helix at the cytoplasmic site of transmembrane domain 1, between the lasso region and transmembrane domain 2. These regions undergo substantial conformational rearrangements initiated by ATP and ligand binding, participating in the switch from the inward‐facing to the outward‐facing conformation of the protein. The p.Arg391 residue is suspected to provide a link between these regions via intramolecular interactions with Glu253, Val1147, and Asn1151. The p.Arg391Gly substitution hinders these interactions (Charbel Issa et al., 2020). Based on these results the aa position 391 might be important in the conformational switch necessary for the molecular function of ABCC6. A second mutation present in just a subset of individuals may further alter the molecular mechanism of the Arg391Gly variant, fully hampering its function and resulting in the emergence of the phenotype. Therefore, we tested whether there was an association of the c.1171A>G (p.Arg391Gly) pathogenic variant with any of the coding SNPs of the *ABCC6* gene in the patients. This analysis did not reveal any c.1171A>G (p.Arg391Gly) associated SNPs in *ABCC6* either among the European or the US patients. We, therefore, concluded that the disease‐causing mechanism of the c.1171A>G (p.Arg391Gly) pathogenic variant is not based on the alteration of an intra‐protein interaction due to the presence of a simultaneous *ABCC6* SNP affecting a second amino acid in the protein.

**Figure 2:**
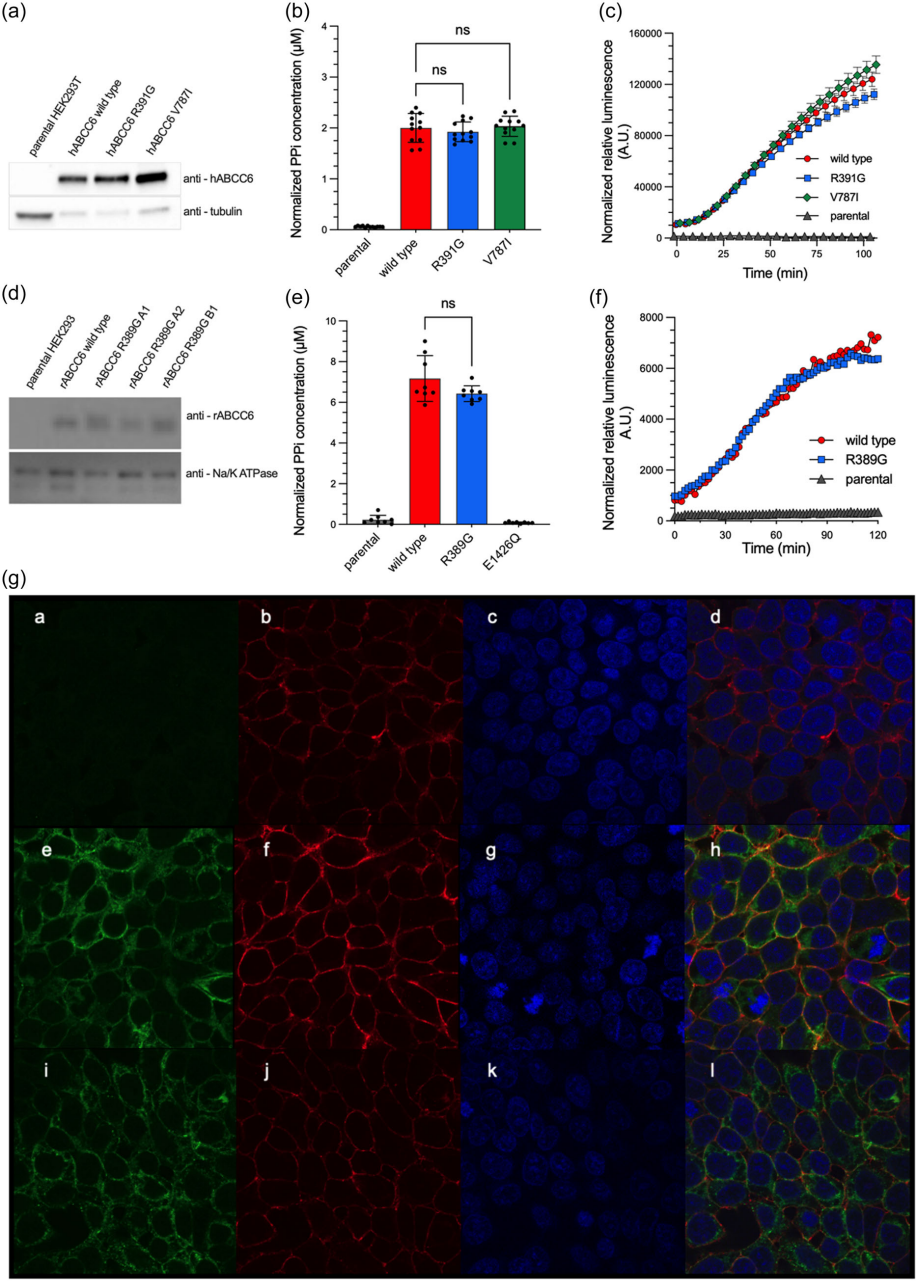
In vitro characterization of the ABCC6 variants. (a) Overexpression of the wild‐type human ABCC6 and the incomplete penetrant variants p.R391G and p.V787I in HEK293T cells, 5 µg protein of total cell lysates shown on Western‐blot. (b) PPi accumulation in the medium of parental HEK293T cells or HEK293T cells overexpressing the wild‐type human ABCC6 or the incomplete penetrant p.R391G and p.V787I variants. Means ± SD are indicated. (c) ATP efflux of parental HEK293T cells or HEK293T cells overexpressing the wild‐type human ABCC6 or the incomplete penetrant p.R391G and p.V787I variants. Means ± SEM are indicated. (d) Overexpression of the wild‐type rat ABCC6 and the p.R389G variant in HEK293 cells. Five microgram protein of total cell lysates shown on Western blot. The A2 clone of rat ABCC6 R389G mutant was selected for further analysis based on its similar expression level compared to that of the wild‐type rat ABCC6. (e) PPi accumulation in the medium of parental HEK293 cells or cells overexpressing the wild‐type rat ABCC6 or the p.R389G and the p.E1426Q catalytically inactive variant. Means ± SD are indicated. (f) ATP efflux of parental HEK293 cells or that of overexpressing the wild‐type rat ABCC6 or the p.R389G and the p.E1426Q catalytically inactive variant. (g) Representative images showing the subcellular localization of the overexpressed wild‐type and p.R389G mutant rat ABCC6 in HEK293 cells by confocal microscopy. Subpanels [a–d] show parental HEK293, [e–h] HEK293 cells overexpressing the wild‐type rat ABCC6 and [i–l] HEK293 cells overexpressing p.R389G mutant rat ABCC6. Green; rat ABCC6, red: plasma membrane marker Na‐K ATPase, blue: Dapi staining.

The lack of any associated SNPs in *cis* or *trans* position ruled out the inter or intramolecular interactions underlying the IP of ABCC6 for p.Arg391Gly and p.Val787Ile. A plausible explanation for the phenomenon is an associated variation in a yet unknown interaction partner (discussed later in the text).

We hypothesized that in vitro, in contrast to the fully penetrant pathogenic mutations, IP variants would behave similarly to wild‐type. Indeed, p.Arg391Gly and p.Val787Ile overexpressed in HEK293T cells showed comparable expression to wild‐type human ABCC6 (Figure 2a). Furthermore, we performed functional analysis of wild‐type hABCC6, which, as expected, when overexpressed, accumulated pyrophosphate (PPi) in the medium of the cells (Figure 2b). Similarly, the IP variants, when overexpressed, also accumulated PPi in the culture media. Pyrophosphate in the conditioned medium is formed from ATP of cellular origin, mainly of ATP efflux mediated by overexpressed ABCC6 (Jansen et al., 2014; Szeri et al., 2020; Szeri et al., 2022). All ATP in the medium is rapidly converted to PPi by ENPP1 in HEK293 cells (Jansen et al., 2014). Thus, PPi levels are proportional to ABCC6‐mediated cellular ATP efflux. HEK293T cells overexpressing IP variants were indistinguishable from those with the wild type. However, PPi concentration in their medium was much higher than in parental (un‐transfected) HEK293T cells (Figure 2b). Furthermore, in direct cellular ATP efflux experiments (Figure 2c), the IP variants were indistinguishable from wild‐type ABCC6, while parental cells showed infinitesimal activity. The rat ABCC6 protein (rABCC6) is significantly more active than the human ortholog, with the region of interest fully conserved (Supporting Information: Figure S1a). HEK293 cells overproducing p.Arg389Gly rABCC6, analogous to p.Arg391Gly in humans, showed similar expression (Figure 2d), PPi accumulation (Figure 2e), and ATP release (Figure 2f) as the wild‐type rABCC6. Correspondingly, plasma membrane localization of the p.Arg389Gly rABCC6 mutant was also similar to that of the wild‐type rABCC6 (Figure 2f). In contrast, cells expressing the catalytically inactive form of rABCC6 (p.Glu1426Gln), despite similar expression levels and intracellular localization (Szeri et al., 2021), were entirely inactive in both functional assays (Figure 2e,f). In conclusion, expression, subcellular localization, and functional activity of IP variants were fully preserved compared to wild type.

PXE displays a remarkable though largely unexplained clinical and molecular heterogeneity. As IP of certain disease‐causing alleles can be a potential mechanism to contribute to this heterogeneity, we sought to determine whether *ABCC6* IP variants could be found in a large European cohort of PXE patients.

To this end, we used a recently created bioinformatic tool with stringent exclusion criteria to avoid artifacts (Mikó et al., 2021). We applied correction for overrepresented homozygous pathogenic variants secondary to inbreeding and, therefore, only 52% of the initial alleles passed the filtering criteria. As a result of enrichment calculations, we found five variants to be more frequent in the reference population than in the patient cohort. Thus we claim that these are non‐pathogenic benign variants although identified as pathogenic previously. Other variants (c.3979G>A (p.G1327R), c.2848G>A (p.A950T), c.3980G>A (p.G1327E), c.1355C>A (p.A452D), c.2095G>A (p.E699K), c.4016G>A (p.R1339H) were enriched in the patient cohort, but not significantly. Only the two latter were present in the US cohort, however, the c.4016G>A (p.R1339H) variant was only represented as a single heterozygous variant. The pathogenicity of all six variants was assessed recently by the Sherloc scoring system and the c.4016G>A (p.R1339H) was predicted pathogenic while the others were classified as likely benign and of unknown significance (Verschuere et al., 2021). However, the population‐genetic approach used herein requires larger well‐characterized cohorts to determine their pathogenicity and penetrance.

We identified two incompletely penetrant variants: c.2359G>A (p.Val787Ile) and c.1171A>G (p.Arg391Gly), with a calculated 6.5% and 1.98% penetrance, respectively. Nevertheless, it should be emphasized that the c.2359G>A (p.Val787Ile) was reported previously in only one patient (Pfendner et al., 2007) and is present solely in two unrelated patients of our European cohort, both originating from France. This variant was absent from the US cohort. Due to the small number of patients harboring this pathogenic variant, it was impossible to compare the severity of its phenotype to the biallelic LOF variant phenotype. The Val787 residue was previously shown to be evolutionarily conserved (Ran et al., 2018). Of note, based on protein glycosylation studies, it has also been suggested recently that the p.Val787Ile ABCC6 has a similar cellular localization as the wild‐type ABCC6 (Ran et al., 2018).

To assess the clinical symptoms of the patients with one c.1171A>G (p.Arg391Gly) pathogenic variant, we made use of the Phenodex scoring (Pfendner et al., 2007). Though the Phenodex scoring is somewhat debated, it is the most widely used tool for the clinical evaluation of PXE. Although the number of patients with c.1171A>G (p.Arg391Gly) was relatively small (*n* = 17), our data showed that, when penetrant, it results in a phenotype as severe as caused by the *ABCC6* LOF variants. This also suggests, that in rare cases (AF_in patients_^2^ = ~1/2500 patients), individuals with homozygous c.1171A>G (p.Arg391Gly) pathogenic variants should also be detected amongst PXE patients. This was indeed recently reported for one patient (Boraldi et al., 2020). However, it should be noted that this patient also suffered from beta‐thalassemia, which was previously shown to lead to a PXE phenocopy in older patients (Hamlin et al., 2003).

There is a further line of evidence supporting the full expressivity of the phenotype caused by the c.1171A>G (p.Arg391Gly) variant. Recessive LOF variants of *ENPP1* lead to generalized arterial calcification of infancy (GACI), a severe form of ectopic calcification, often leading to perinatal lethality (Ferreira et al., 2021). Strikingly, several patients with GACI phenotype have biallelic pathogenic variants in *ABCC6* in the absence of any pathogenic *ENPP1* variants (Nitschke et al., 2012). Importantly, there were GACI patients diagnosed with c.1171A>G (p.Arg391Gly) ABCC6 in different clinical centers (Li et al., 2014; Nitschke et al., 2012). Although the c.1171A>G (p.Arg391Gly) variant has an AF of 0.78% in the general population, it is very unlikely that among the 34 unrelated *ABCC6‐*related GACI patients, three patients would carry the c.1171A>G (p.Arg391Gly) variant only by chance. This further confirms that in the appropriate context, the c.1171A>G (p.Arg391Gly) variant has full expressivity.

The low penetrance of c.1171A>G (p.Arg391Gly) also suggests that in the gnomAD database containing genomic data from healthy individuals homozygous c.1171A>G (p.Arg391Gly) variants are expected at a theoretical frequency of 6.1/100,000 since the AF is 0.78% in that population. Indeed, there are two homozygous healthy individuals (both >40 years) among the 63124 European individuals in the gnomAD database, which is not different from the expected frequency (*p* = 0.58). Furthermore, we have recently found an individual homozygous for c.1171A>G (p.Arg391Gly) without any PXE‐related phenotype. However, these observations should be taken with caution since it is not completely excluded that patients with non‐congenital, late‐onset diseases may also be included in gnomAD (Karczewski et al., 2020). Additionally, it has been shown recently that in some rare cases a very mild, eye‐restricted form of PXE can develop in the elderly harboring the c.1171A>G (p.Arg391Gly) pathogenic variant (Charbel Issa et al., 2020), which most probably remains undetected in most of the cases.

Trying to find an explanation for the IP at the molecular level, we first ruled out the association of any specific *ABCC6* variants either in *cis* (same allele) or *trans* (other allele) with c.1171A>G (p.Arg391Gly). We could not exclude the potential role of deep intronic variants. However, we also verified the available noncoding intronic variants, but none of them were systematically associated with the c.1171A>G (p.Arg391Gly) in the patients. As we did not identify any other variant associated with *ABCC6* in PXE patients, we thought that the molecular mechanism of IP is different. We hypothesized that the c.1171A>G (p.Arg391Gly) variant becomes pathogenic if ABCC6 participates in a protein‐protein interaction that is crucial for the correct localization/function of ABCC6. Such an interaction partner has not yet been identified for ABCC6. However, in the ABCC gene family two proteins, SUR1 and SUR2, are ion channel regulators physically interacting with other membrane proteins (Ding et al., 2019; Foster & Coetzee, 2016). It has also been shown that CFTR interacts with several other proteins (Haggie et al., 2006; Ko et al., 2004). According to our hypothesis, the interaction between ABCC6 and the unidentified partner protein takes place through the p.Arg391 residue, which is in a highly conserved amino acid stretch, strengthening our hypothesis on its critical role (Supporting Information: Figure S1). We hypothesize that this interaction with the unknown protein would be ablated by the presence of the c.1171A>G (p.Arg391Gly) variant only in the case when the interacting partner also has a rare sequence variant, unable to interact with p.Arg391Gly but still functional with the p.Arg391 residue. According to this theory, for the elimination of the interaction between the ABCC6 Arg391 and the other protein, both alleles encoding for the interacting protein should be mutated, which would explain the very low penetrance of the c.1171A>G (p.Arg391Gly) variant. A similar mechanism could explain the IP of the c.2359G>A (p.Val787Ile) variant.

It should be emphasized that the putative interaction between ABCC6 and the yet‐unknown partner may occur at various levels. This interaction might take place temporarily during protein maturation or trafficking, affecting the localization, stability, turnover, or the plasma membrane retention of ABCC6. Last, but not least, it may as well directly affect the primary physiological function of ABCC6, mediating cellular ATP efflux. Interestingly, an important proportion of ABCC6 missense variants are localization mutants, which retain their enzymatic activity if their plasma membrane localization is rescued. Hypothesizing that the interaction partner of ABCC6 is related to the localization of ABCC6 might even give hints about potential therapies with chemical chaperones, for example, 4‐phenyl‐butyrate (4‐PBA), an FDA‐approved drug. Indeed, 4‐PBA was already shown to rescue some mis‐localized ABCC6 mutants retained in the ER (Pomozi et al., 2017; Saux Le et al., 2011).

Collectively, our data have important clinical relevance for genetic counseling. First, the c.1171A>G (p.Arg391Gly) pathogenic variant can cause either PXE or GACI. Second, the expected clinical phenotype is not different from any other known pathogenic variants leading to these diseases. Third, and most importantly, based on our study this pathogenic variant has low penetrance, which probably relies on sequence variants in other gene(s), thus the co‐occurrence of c.1171A>G (p.Arg391Gly) dependent disease in siblings is probably much less frequent than expected. Along these lines, it seems reasonable to consider pathogenic variants of low or very low penetrance (e.g., c.2359G>A (p.Val787Ile)) as risk factors, in the general sense. The identification of the mechanism underlying penetrance will be crucial to provide correct pre‐conceptional counseling to couples where one or both partners carry the c.1171A>G (p.Arg391Gly) allele.

Our experimental data opens new avenues in PXE research for the identification of interacting partner(s), which might become important biomarker(s) and pharmacological target(s) in PXE or other ectopic calcification disorders.

## CONFLICT OF INTEREST

The authors declare no conflict of interest.

## Supporting information

Supplementary Figure 1. (A) Alignment of the sequence flanking the R391 amino acid residue in various vertebrates. The region adjacent to R391 of ABCC6 is highly conserved throughout evolution. R391 is in bold, to highlight its evolutionary conservation in all species analyzed from human to zebrafish and electric eel. Of note, in the two latter species there are two ABCC6 genes. (B) Alignment of the sequence flanking the ABCC6 R391 amino acid residue (in bold) in various members of the ABCC family.

Supplementary information on Methods in Supplementary File 2.

Supplementary Table 1: Genetic and clinical characterization of 589 European PXE patients

## Data Availability

Data sharing not applicable to this article as no datasets were generated or analyzed during the current study.
