# Supplementary material for: The pathogenic c.1171A>G (p.Arg391Gly) and c.2359G>A (p.Val787Ile) ABCC6 variants display incomplete penetrance causing pseudoxanthoma elasticum in a subset of individuals
Source: Hum Mutat. 2022 Nov 15;43(12):1872–81. doi: 10.1002/humu.24498 (PMC9772137; doi:10.1002/humu.24498)

Supplementary Figure 1. (A) Alignment of the sequence flanking the R391 amino acid residue in various vertebrates. The region adjacent to R391 of ABCC6 is highly conserved throughout evolution. R391 is in bold, to highlight its evolutionary conservation in all species analyzed from human to zebrafish and electric eel. Of note, in the two latter species there are two ABCC6 genes. (B) Alignment of the sequence flanking the ABCC6 R391 amino acid residue (in bold) in various members of the ABCC family.


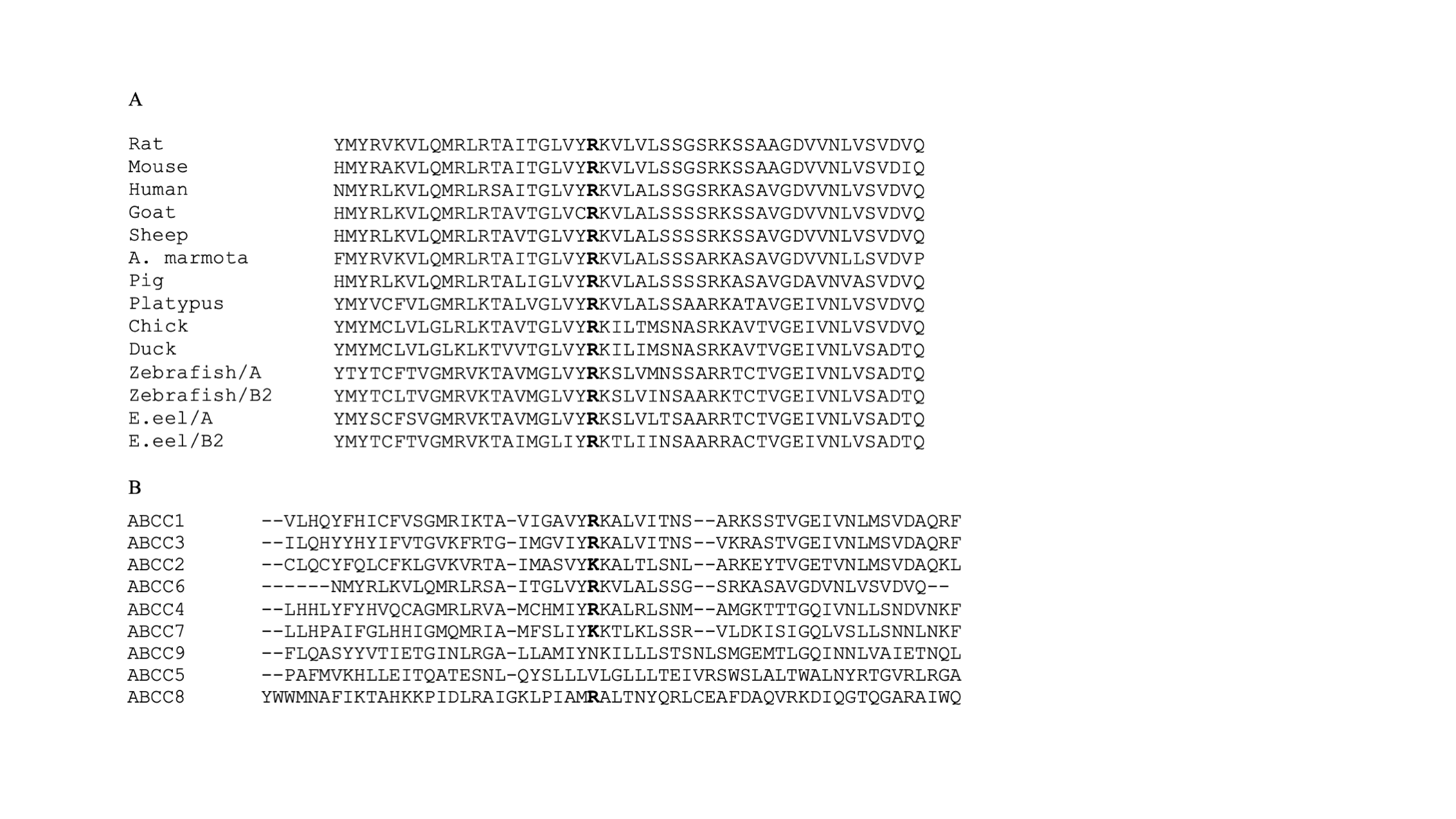

Supplement: Supplementary file 1 — Supplementary Figure 1. (A) Alignment of the sequence flanking the R391 amino acid residue in various vertebrates. The region adjacent to R391 of ABCC6 is highly conserved throughout evolution. R391 is in bold, to highlight its evolutionary conservation in all species analyzed from human to zebrafish and electric eel. Of note, in the two latter species there are two ABCC6 genes. (B) Alignment of the sequence flanking the ABCC6 R391 amino acid residue (in bold) in various members of the ABCC family. [file HUMU-43-1872-s003.docx]
