# Supplementary material for: The pathogenic c.1171A>G (p.Arg391Gly) and c.2359G>A (p.Val787Ile) ABCC6 variants display incomplete penetrance causing pseudoxanthoma elasticum in a subset of individuals
Source: Hum Mutat. 2022 Nov 15;43(12):1872–81. doi: 10.1002/humu.24498 (PMC9772137; doi:10.1002/humu.24498)
Supplement: Supplementary file 3 — Supplementary Table 1: Genetic and clinical characterization of 589 European PXE patients [file HUMU-43-1872-s001.docx]

## Patients

All PXE patients signed an informed consent. The study was approved by the University Hospital of Angers, the Ethics Committee of Ghent University Hospital, and of the University of Modena and Reggio Emilia. The Declaration of Helsinki was followed. The US patients were recruited under the IRB of Thomas Jefferson University, PA and the Genetic Alliance IRB – protocol PXE #001. Altogether 397 female and 191 male patients were included in the European cohort. The international US cohort was composed of 478 patients (Saeidian et al. 2022). Patients were clinically diagnosed as described earlier (Vanakker et al. 2008; Pfendner et al. 2007; Boraldi et al. 2021) and mutation detection was performed as described below.

## Bioinformatic analysis

Bioinformatic analysis was performed by the population-genetic based algorithm as described elsewhere (Mikó et al. 2021). Briefly, the unified European patient cohorts underwent filtering (patients with familial relationship, alleles with large deletion >50 were excluded). We also removed alleles when the patient’s other allele was filtered out and performed correction for inbreeding. Next we determined variant enrichment analysis in the filtered patient cohort relative to the European non-Finnish population of the gnomAD database to confirm pathogenicity by comparing allele frequency (AF). Significantly enriched alleles (Fisher-exact test) were tested for IP. For this, the enrichment of each significantly enriched variant was compared to the cumulative enrichment of fully penetrant complete loss-of-function (LOF) sequence variants by Fisher-exact test. The ratio of the enrichment of the specific variant (V) and that of the LOF variants (V/LOF ratio) provides a good estimate of the penetrance (Mikó et al. 2021). We considered the variant incompletely penetrant if the enrichment of V and LOF were significantly different after Bonferroni correction for multiple testing.

***Mutation detection***

Mutation detection was performed as described earlier. To distinguish pseudogenes from the *ABCC6* gene and determine large deletions (Pulkkinen et al. 2001; Pfendner et al. 2007; Symmons, Váradi, and Arányi 2008). Briefly, genomic DNA was isolated from whole blood (QIAamp blood kit, Qiagen®) according to the manufacturer’s protocol. Molecular analysis of the coding sequence and the intron-exon boundaries of the *ABCC6* gene was performed as previously described (Vanakker et al. 2008; Hosen et al. 2015). C-notations are based on NM_001171.5 and p-notations on NP_001162.4. MLPA analysis of the *ABCC6* gene was performed to evaluate the presence of larger deletions or insertions by using commercially available SALSA MLPA kit PO92-B3 (MRC-Holland) and according to the manufacturer’s recommendations ([www.mlpa.com](http://www.mlpa.com)).

***Mutagenesis***

Mutagenesis was performed as described previously (Szeri et al. 2021; Szeri, Niaziorimi, et al. 2020). Briefly, mutations were introduced into Gateway entry vectors pEntr223-rAbcc6 and pEntr223-hABCC6 by uracil-specific excision reagent (USER) cloning with the primers listed in Supplementary Table 2 using Phusion U PCR master mix (Thermo Scientific, Waltham, MA, USA).

| mutant  construct | forward primer sequence 5' to 3' | reverse primer sequence 5' to 3' |
| --- | --- | --- |
| rAbcc6  R389G | ACCTTTCCGUACACCAGGCC  AGTGATGGC | ACGGAAAGGUCCTGGTCCTGTCCAG  TGGTTCCA |
| hABCC6  R391G | ACGGAAAGGUCCTGGCTCTG  TCCAGCGG | ACCTTTCCGUACACCAGGCCAGTGATGGCCG |
| hABCC6  V787I | ATGCCCACAUTGGCCAGCAT  GTCTTCAACCAGGTCATT | ATGTGGGCAUCCAGGGCCGCCAGGGG |

Supplementary Table 2: Primer pairs used to generate site-specific mutations in the cDNA encoding the rat and human ABCC6.

PCR fragments were purified using Nucleospin gel and PCR cleanup kit (Macherey-Nagel, Düren, Germany) and assembled using USER enzyme mix (New England Biolabs, Ipswich, MA, USA), according to the manufacturer’s instructions. Resulting circular constructs were verified by Sanger sequencing with the respective primers listed in Supplementary Table 3 and transformed into competent E. coli DH5alpha cells.

| primer name | primer sequence 5' to 3' |
| --- | --- |
| M13 Fwd | TGTAAAACGACGGCCAG |
| hABCC6 Fwd 1 | CGCCTGAGGCCCCAGAATTCCT |
| hABCC6 Fwd 2 | CGGAGGCACAACAAGGCAATAGCA |
| hABCC6 Fwd 3 | TGTGGCTGCCTCTCGTCTGGAT |
| hABCC6 Fwd 4 | CCCGGGTGTCCTTTGACCGTCT |
| hABCC6 Fwd 5 | CCTGGCCCGGGCTGTATACAGA |
| hABCC6 Fwd 6 | CCAATACGGCAGGGTGAAGGCC |
| hABCC6 Fwd 7 | ACGCTGGGTTTCAGAGCCTGTA |
| hABCC6 Fwd 8 | GCGGGCAGATCGAGTTCCAGGA |
| hABCC6 Fwd 9 | TCCTGGACGAGGCTACTGCTGC |
| rAbcc6 Fwd1 | CTCACCACCATGAGCTTCG |
| rAbcc6 Fwd2 | CAGAAGAACTTGTTTCCCAGC |
| rAbcc6 Fwd3 | GGCCTGGTGTACAGAAAGGT |
| rAbcc6 Fwd4 | GCTAGGTGCCCTGAAGACCT |
| rAbcc6 Fwd5 | GCTGTCTGCTGGCTGTTGT |
| rAbcc6 Fwd6 | CAGCCAGGAAGTCTTCAAACA |
| rAbcc6 Fwd7 | CAGGAGAGGACAGTGTGCAG |
| rAbcc6 Fwd8 | CGGACATAGTGGATGTGGAC |
| rAbcc6 Fwd9 | CGCTACATGTGCTGTGCTG |
| rAbcc6 Fwd10 | GTGGTATTTGGATCGATGGG |
| rAbcc6 Fwd11 | CAGTGCTGCTCATTGCTCAC |

Supplementary Table 3: Sequencing primers used in Sanger sequencing to confirm the DNA sequence of human ABCC6 and rat Abcc6 mutants.

The cDNAs encoding pEnter223-rAbcc6 and pEntr223-hABCC6 mutants were subsequently subcloned into a Gateway compatible pQCXIP expression vectors using LR Clonase-II (Thermo Scientific, Waltham, MA, USA)

***Cell culturing and generation of mutant cell lines***

Cell culturing and generation of mutant cell lines were performed as described previously (Szeri et al. 2021; Szeri, Niaziorimi, et al. 2020). Briefly, HEK293 and HEK293T cells were cultured at 37^o^C and 5% CO_2_ in humidifying conditions in HyClone DMEM (GE) completed with 100 units pen/strep per ml (Gibco) and 5% FBS. pQCXIP expression vectors containing the cDNA of the wild type and mutant hABCC6 and rAbcc6 were transfected to HEK293T and HEK293 cells, respectively, with calcium phosphate precipitation method. The transfected cells were selected in completed DMEM medium also containing 2 µM puromycin (Gibco). Cell lines were established from clones showing high expression of the respective hABCC6 and rAbcc6 mutants. Of note, several clones were generated for each mutation and these subclones behaved very similarly with respect to PPi accumulation in the culture medium.

## Immunoblot and analysis of rat ABCC6

After puromycin selection the expression of the hABCC6 R391G, hABCC6 V787I and rat Abcc6 R389G in isolated cell clones was confirmed by immunoblot analysis and compared to that of the respective wild-type human or rat ABCC6 overexpressing cell lines. Cell lysates were prepared in lysis buffer (10 mM KCl, 10 mM Tris-HCl and 1.5 mM MgCl_2_, pH 7.4) supplemented with protease inhibitors (Roche). Samples containing 5 µg of total protein determined by BCA assay (Pierce™ BCA Protein Assay Kit, Thermo Scientific) were separated on 7.5% SDS-polyacrylamide gels (Bio-Rad) and transferred to a PVDF membrane with a semi-dry system (Bio-Rad). Wild-type and mutant human ABCC6 were detected with M6II-7 rat anti-human ABCC6 antibody (sc-57528, Santa Cruz Biotechnology) diluted by 1:500 and HRP-conjugated rabbit anti-rat secondary antibody (A9542, Sigma Aldrich) diluted by 1:5000. Wild-type and mutant rat Abcc6 were detected with the polyclonal K14 rabbit anti-rat ABCC6 antibody diluted by 1:3000 (kind gift of Dr. Bruno Stieger and HRP-conjugated donkey anti-rabbit secondary antibody (SA1200, Fisher Scientific) in a 1:5000 dilution.

For loading controls we used mouse anti- α-tubulin antibody (T6199, Sigma Aldrich) in a 1:1000 dilution, and HRP-conjugated rabbit anti-mouse secondary antibody (A9044, Sigma Aldrich) in a 1:10.000 dilution; and mouse anti-alpha 1 sodium potassium ATPase antibody (ab7671, Abcam) in an XXX dilution and HRP-conjugated XXX anti-mouse secondary antibody (XXX).

The signal was visualized by ECL (Pierce Western blotting substrate, Thermo Scientific).

## Subcellular localization of rat ABCC6 in HEK293 cells

Rat ABCC6 was detected as described previously (Szeri et al. 2021; Szeri, Niaziorimi, et al. 2020). In short, HEK293 cells were seeded and grown for 2 days on ibi-Treat 1.5 µ-Slide 4 well chamber slides (80426, Ibidi) previously coated with poly-D-lysine. Cells were fixed in 4% PFA and subsequently in -20 ºC cold methanol for 5 min each. Samples were blocked with Protein Block solution (BioGenex) for 60 min. Coverslips were incubated with the polyclonal rabbit anti-rat ABCC6 antibody K14 diluted by 1:100 (kind gift of Dr. Bruno Stieger) and the mouse monoclonal anti-alpha 1 sodium potassium ATPase antibody (ab7671, Abcam) diluted by 1:250 for 60 min. Subsequently, samples were incubated with A488-conjugated anti-rabbit secondary antibody (A11008, Fisher Scientific) and A568 conjugated anti-mouse antibody (A11004, Fisher Scientific) both diluted by 1:1000 for 60 min. Samples were subsequently incubated with 300nM DAPI (40043, Biothium) for 5min to stain nuclei. The intracellular localization of the wild type and mutant rat ABCC6 were analysed by two point-scanning laser confocal microscope Nikon Eclipse T*i* equipped with a Nikon A1R+ at the Bioimaging Shared Resource of the Sidney Kimmel Cancer Center (NCI 5 P30 CA-56036).

## Quantification of PP_i_ levels in the medium of cells

Functional assessment of cell lines was performed as described previously (Szeri et al. 2021; Szeri, Niaziorimi, et al. 2020). Briefly, for the PPi accumulation assays HEK293 or HEK293T cell lines were seeded in 96-well plates in completed DMEM, and experiments were conducted with wells using cells forming confluent monolayers (Szeri et al. 2021; Szeri, Niaziorimi, et al. 2020). Confluent HEK293 and HEK293T cells in 96-well plates were incubated in 100µl fresh medium for 24 hours. PP_i_ concentration of the medium samples were determined as described previously (Szeri, Lundkvist, et al. 2020). First, PPi was converted into ATP in an assay containing 50 mM HEPES pH 7.4, 80 µM MgCl_2_, 32 mU/ml ATP Sulfurylase (New England Biolabs) and 16 µM adenosine 5´-phosphosulfate (Sigma-Aldrich) by incubating samples and standards at 37˚C for 30 min followed by inactivation of the enzyme at 90˚C for 10 minutes. In a consecutive step, ATP content was determined in a bioluminescent assay adding BacTiterGlo (Promega,) to samples and standards in a 1:1 ratio. PPi concentration of plasma samples was calculated with standard calibration. Values were corrected for the initial sample ATP concentrations. Data were normalized for expression levels based on Western blots quantified with ImageLab 6.1.0 (Bio-Rad) and for cell metabolic activity applying 5% PrestoBlue Cell Viability Reagent (A13262, Invitrogen) to cells after the measurements and determining fluorescence at 545nm/585nm in an EnSpire Multimode Plate Reader (PerkinElmer) in white 96 well Optiplates (6005290, PerkinElmer).

## Real-time ATP efflux assay

Real-time ATP efflux assay were conducted as described previously (Szeri et al. 2021; Szeri, Niaziorimi, et al. 2020). HEK293 or HEK293T cells seeded in poly-D-lysine-coated black 96-well plates with an optically clear bottom (165305, Thermo Scientific) were allowed to grow to confluence in 2 days in completed DMEM medium. At confluency the medium was removed and replaced by 50 μl efflux buffer, consisting of 11.5 mM HEPES (pH 7.4), 130 mM NaCl, 5 mM MgCl_2_, 1.5 mM CaCl_2_ and 11.5 mM glucose. Cells were incubated for 1 hour at 27 ºC in efflux buffer. Next, 50 µl efflux buffer containing 10% BactiterGlo (Promega) reactant previously dissolved in efflux buffer according to the instructions of the manufacturer was added to each well. Bioluminescence was subsequently determined in real time in a Flex Station 3 microplate reader (Molecular Devices) or in an EnSpire Multimode Plate Reader (PerkinElmer). The real-time ATP efflux assay was run at 27 ºC for the first 1 hour and then at 37 ºC for 2 hours (readings at 37˚C are depicted in the figures). The initial low temperature allowed the endogenous ectonucleotidases to metabolize the excess ATP generated by medium-change initiated sheer-stress resulting to an ABCC6-independent ATP efflux, that otherwise gave a significant background in the experiments.

Data were normalized for expression levels based on Western blots quantified with ImageLab 6.1.0 (Bio-Rad) and for cell metabolic activity applying 5% PrestoBlue Cell Viability Reagent (A13262, Invitrogen) to cells after the measurements and determining fluorescence at 545nm/585nm in an EnSpire Multimode Plate Reader (PerkinElmer).

## Statistical analyses

Data were analyzed using Prism 9.3.1 (GraphPad Software Inc.). Correlation between age and Phenodex score was assessed by linear regression with 95% confidence intervals. One-way ANOVA was applied to test significance of pyrophosphate concentrations of HEK293T cells overexpressing the wild type and the p.R391G or the p.V787I variants. Two-tailed t-test was applied to test significance of pyrophosphate concentrations of HEK293 cells overexpressing the wild type or the p.(R391G) variant. Significance was accepted at p<0.05.

References

Hosen, MJ., Van Nieuwerburgh, F., Steyaert, W., Deforce, D., Martin, L., Leftheriotis, G., De Paepe, A., Coucke, PJ., Vanakker, and OM. 2015. "Efficiency of exome sequencing for the molecular diagnosis of pseudoxanthoma elasticum." *The Journal of investigative dermatology* 135 (4). <https://doi.org/10.1038/jid.2014.421>. <https://www.ncbi.nlm.nih.gov/pubmed/25264593>.

Pfendner, EG, O.M. Vanakker, S. Terry, S. Vourthis, P.E. McAndrew, M.R. McClain, S. Fratta, A.S. Marais, S. Hariri, P.J. Coucke, M. Ramsay, D. Viljoen, P.F. Terry, A. De Paepe, J. Uitto, and L.G. Bercovitch. 2007. "Mutation Detection in the ABCC6 Gene and Genotype-Phenotype Analysis in a Large International Case Series Affected by Pseudoxanthoma Elasticum." *Journal of medical genetics* 44 (10). <https://doi.org/10.1136/jmg.2007.051094>. <https://www.ncbi.nlm.nih.gov/pubmed/17617515>.

Pulkkinen, L., A. Nakano, F. Ringpfeil, and J. Uitto. 2001. "Identification of ABCC6 Pseudogenes on Human Chromosome 16p: Implications for Mutation Detection in Pseudoxanthoma Elasticum." *Human genetics* 109 (3). <https://doi.org/10.1007/s004390100582>. <https://www.ncbi.nlm.nih.gov/pubmed/11702217>.

Symmons, O., A. Váradi, and T. Arányi. 2008. "How Segmental Duplications Shape Our Genome: Recent Evolution of ABCC6 and PKD1 Mendelian Disease Genes." *Molecular biology and evolution* 25 (12). <https://doi.org/10.1093/molbev/msn202>. <https://www.ncbi.nlm.nih.gov/pubmed/18791038>.

Szeri, F., V. Corradi, F. Niaziorimi, S. Donnelly, G. Conseil, S. P. C. Cole, D. P. Tieleman, and K. van de Wetering. 2021. "Mutagenic Analysis of the Putative ABCC6 Substrate-Binding Cavity Using a New Homology Model." *Int J Mol Sci* 22 (13). <https://doi.org/10.3390/ijms22136910>. <https://www.ncbi.nlm.nih.gov/pubmed/34199119>.

Szeri, F., S. Lundkvist, S. Donnelly, U.F.H. Engelke, K. Rhee, C.J. Williams, J.P. Sundberg, R.A. Wevers, R.E. Tomlinson, R.S. Jansen, and K. van de Wetering. 2020. "The Membrane Protein ANKH Is Crucial for Bone Mechanical Performance by Mediating Cellular Export of Citrate and ATP." *PLoS genetics* 16 (7). <https://doi.org/10.1371/journal.pgen.1008884>. <https://www.ncbi.nlm.nih.gov/pubmed/32639996>.

Szeri, F., F. Niaziorimi, S. Donnelly, J. Orndorff, and K. van de Wetering. 2020. "Generation of fully functional fluorescent fusion proteins to gain insights into ABCC6 biology." *FEBS Lett*. <https://doi.org/10.1002/1873-3468.13957>. <https://www.ncbi.nlm.nih.gov/pubmed/33058196>.

Vanakker, OM., Leroy, BP., Coucke, P., Bercovitch, LG., Uitto, J., Viljoen, D., Terry, SF., Van Acker, P., Matthys, D., Loeys, B., De Paepe, and A. 2008. "Novel clinico-molecular insights in pseudoxanthoma elasticum provide an efficient molecular screening method and a comprehensive diagnostic flowchart." *Human mutation* 29 (1). <https://doi.org/10.1002/humu.9514>. <https://www.ncbi.nlm.nih.gov/pubmed/18157818>.
